# Foundation doctor knowledge of wounds and dressings is improved by a simple intervention: An audit cycle-based quality improvement study

**DOI:** 10.1016/j.amsu.2020.01.004

**Published:** 2020-01-21

**Authors:** Hannah Catton, Luke Geoghegan, Alexander J. Goss, Raina Zarb Adami, Jeremy N. Rodrigues

**Affiliations:** a Stoke Mandeville Hospital, Aylesbury, UK; b Academic Section of Vascular Surgery, Department of Surgery and Cancer, Imperial College London, London, UK; c Royal Free NHS Trust, London, UK; d Addenbrooke's Hospital, Cambridge, UK; e Nuffield Department of Orthopaedics, Rheumatology and Musculoskeletal Sciences (NDORMS), University of Oxford, Oxford, UK

**Keywords:** Wound, Wounds and injuries, Dressings, Education

## Abstract

**Background:**

Many foundation year 1 and 2 doctors (FYs) have limited knowledge experience in wound management. Wound dressing formularies exist in many NHS Trusts, though awareness of and adherence to them by FYs is not known. This quality improvement study described baseline FY knowledge of wound management, and investigated whether this could be improved through educational intervention.

**Methods:**

A single-centre, prospective, baseline audit was conducted following local approval. This assessed knowledge of wound types and appropriate dressings alongside individual confidence providing wound care. The educational intervention involved the distribution of an ID-badge sized quick reference guide that could be attached to the FYs' lanyards, and an introduction to the formulary during routine teaching. The audit loop was closed by repeating the questionnaire.

**Results:**

Pre- (n = 43) and post- (n = 35) intervention questions were completed by FYs. The mean score post-intervention was significantly higher than the pre-intervention score across all knowledge questions (from 32% correct to 71% correct, p < 0.0001). There was no change in participant confidence, which remained low.

**Conclusion:**

FYs lack confidence and knowledge about wounds and dressings. The latter can be improved through a simple and practical educational intervention that could be deployed nationally.

## Highlights

• Junior doctors lack confidence and knowledge on wounds and wound care.
• Knowledge on appropriate wound care can be improved by a simple educational intervention.
• Junior doctor confidence managing wounds is not improved by educational interventions alone.

## 1 Introduction

Wounds pose a large burden to both patients and healthcare systems. More than one in four patients in an average district general hospital a require specialist wound care, with more than 40% undergoing daily dressing changes [1].

Being able to appropriately examine wounds and select suitable treatments are vital aspects of care, which influence the clinical course of wound healing and the development of wound related morbidity [2,3]. Appropriate wound management in the acute setting may promote wound healing and have the additive effect of reducing wound-related complications [4].

A significant proportion of ward-based wound assessment and decision-making is provided by junior doctors, such as Foundation Year doctors (FYs), comprising Foundation Year 1s (FY1s) and Foundation Year 2s (FY2s). However, as there is limited exposure to plastic surgery in UK medical schools’ undergraduate curricula [5], most are unlikely to have received formal medical training in wound management.

Many National Health Service (NHS) Trusts have developed Wound dressing formularies (WDFs) [6]. These aim to standardise dressing use, but also provide an opportunity to demystify wound assessment and dressing choice for clinical professionals. However, it is not known whether FYs have awareness of such formularies, and if they do, whether they adhere to them. Previous work has demonstrated poor understanding of wound management principles in the post-operative setting [7], however, the dissemination of wound care guidelines in the form of a handbook has been shown effectively rationalise dressing choice and ensure regional consistency amongst district nurses when providing wound care in the community [8]. Currently, there is little evidence surrounding non-specialist doctors’ understanding of wound types and dressings. The aim of this quality improvement study was to establish whether FYs have adequate knowledge of wound assessment and dressing choices, and assess whether this could be improved through an audit cycle based around a simple, applicable and reproducible educational intervention.

## 2 Methods

In keeping with national guidance from the Health Research Authority, and with the UK Policy Framework for Health and Social Care Research, this study was classified as clinical audit. Clinical audit in the UK NHS is formally exempt from ethical approval, and such projects are not submitted to an IRB [9]. The project was registered with the Clinical Audit department at Buckinghamshire Healthcare NHS Trust, who confirmed this classification and registered the project on a trust specific intranet system (reference: 4711) and has been registered retrospectively on Research Registry (identification number: researchregistry5314, https://www.researchregistry.com/browse-the-registry#home/registrationdetails/5e14bc3ff162bf0017a1f463). After local approval was obtained, a single centre prospective quality improvement project was conducted in October 2015. The Revised Standards for Quality Improvement Reporting Excellence (SQUIRE 2.0) guidelines were used in the reporting of the present quality improvement study [10].

### 2.1 Study population

All participants sampled were at foundation years 1 and 2 of training across a range of medical and surgical specialties. Participants were identified at mandatory teaching sessions to ensure consistency amongst studied cohorts.

### 2.2 Questionnaire design

A questionnaire comprising multiple-choice items was developed using the Buckinghamshire Healthcare NHS Trust WDF [11]. Table 1 outlines the formulary's recommendation of dressing for different wound types presented in the questionnaire.

**Table 1:** Wound types and appropriate dressings outlined in the Buckinghamshire Healthcare Trust Wound Dressing Formulary.

Wound type	Dressing type	Exemplar product
Epithelialising	Non-adherent	Atrauman™
	Hydrocolloid	Duoderm™
Granulating	Non-adherent	Atrauman™
	Hydrocolloid	Duoderm™
Sloughy	Hydrogel	Purilon gel™
Infected	Iodine/silver-based	Aquacel Ag™

The questionnaire items assessed recognition of wound types in four items and appropriate dressings for those wound types in the other four items. A further two items examined self-assessed confidence with wound care and familiarity with the formulary. Questionnaires were scored based on appropriate management outlined in the Buckinghamshire Healthcare Trust WDF. The same questionnaire was used in the baseline audit and the re-audit to close the audit loop.

### 2.3 Intervention and measures

As a non-clinical medical education project, prospective approval was granted by the local postgraduate tutor. The educational intervention involved discussion of the WDF using pre-existing ward posters and a structured teaching session. This was consolidated by distributing an ID-badge sized quick reference guide that could be attached to junior doctors’ lanyards. The study intervention was deployed immediately after the completion of the pre-intervention questionnaire. The re-audit was conducted two weeks after the intervention.

### 2.4 Statistical analysis

Pre- and post-intervention scores were evaluated using paired t-tests with significance set at p < 0.05. Statistical analysis was performed using SPSS v24.0 (IBM Corp. NY, USA).

## 3 Results

### 3.1 Participants

Forty-three participants were audited pre-intervention. Two further participants were excluded as their attendance was interrupted by clinical duties and so they were not able to complete the questionnaire. The participants comprised 28 FY1s and 15 FY2s. Thirty-five of the pre-intervention participants were available and completed the post-intervention part of the audit cycle. This latter group comprised 23 FY1s and 12 FY2s. For the analysis, the percentage of questions examining junior doctor knowledge answered correctly was calculated. There was no significance difference between FY1s′ scores and FY2s’ scores, either pre-intervention of post-intervention (p = 0.53 and p = 0.45 respectively), so the pre- and post-intervention cohorts were analysed together irrespective of year of training.

### 3.2 FY knowledge

**Fig. 1:**
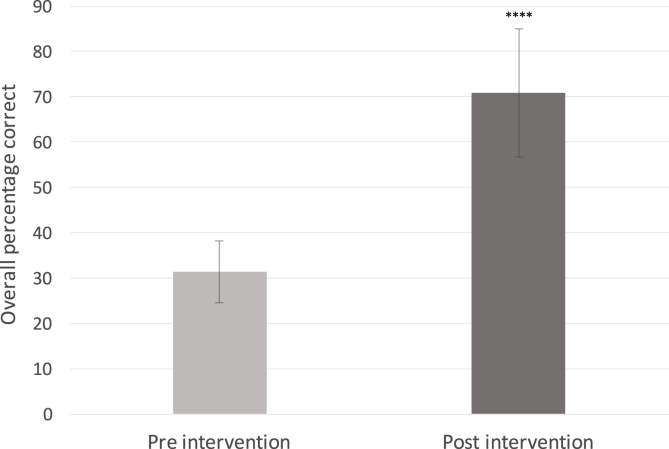
Change in overall percentage score before and after educational intervention (Mean ± SD). ****p < 0.0001, Student's *t*-test.

The mean post-intervention score (70%, SD 26%) was significantly higher than the mean pre-intervention score (32%, SD 22%), (p < 0.0001), see Fig. 1.

**Fig. 2:**
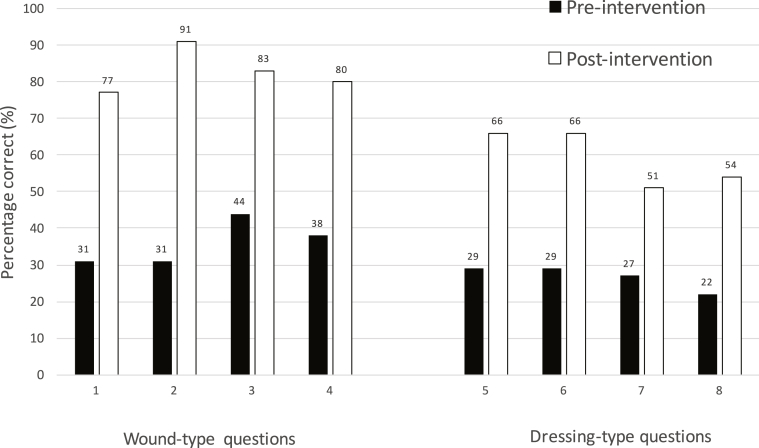
Graph demonstrating the improvement pre- and post-intervention for each item.

Candidate performance increased across all questions post-intervention, as outlined in Table 2. Improvements were seen for answers to questions on both wound types and on dressings. The shift in performance resulting from the intervention is displayed in Fig. 2.

**Table 2:** Breakdown of questions and candidate performance both pre- and post-intervention.

Question Number	Pre-intervention (n = 43)		Post-intervention (n = 35)		% Improvement
	Actual correct	% correct	Actual correct	% correct	
1	14	31	27	77	**+148%**
2	13	29	23	66	**+128%**
3	14	31	32	91	**+194%**
4	13	29	23	66	**+128%**
5	20	44	29	83	**+89%**
6	12	27	18	51	**+89%**
7	17	38	28	80	**+111%**
8	10	22	19	54	**+145%**
All questions	32%		70%		**+119%**

### 3.3 FY confidence

Pre-intervention, no participants felt comfortable to recommend dressings for wounds. This remained unchanged after the intervention, despite the increase in knowledge demonstrated. Ninety-four percent reported that they did not feel comfortable with their level of knowledge. Pre-intervention, 56% of participants reported that increased awareness of the WDF would be beneficial. Post-intervention, this figure rose to 83% of participants.

## 4 Discussion

This study demonstrates the feasibility and efficacy of deploying an educational intervention to improve junior doctor knowledge of wounds and dressings, by providing targeted access to the WDF. Overall, this achieved a significant improvement in the ability of FYs to correctly identify different wound types and select appropriate dressings. The intervention chosen was structured deliberately, so that it could easily be accommodated into the existing mandatory junior doctor teaching programme. Furthermore, the format of the quick reference guide used is simple, cheap and reproducible. This can easily be adapted to other healthcare systems, using local wound management algorithms. As well as suggesting that such an intervention may improve the care patients receive, the data from the audit loop completion also suggest that FYs gave increased value to the WDF after the intervention. Thus, interventions like this may facilitate local implementation of clinical guidance.

Despite these effects, a concurrent improvement in FY confidence was not demonstrated, with all participants remaining uncomfortable with their knowledge of wound types and appropriate dressings. Further investigation of this is needed but given how commonly wounds are encountered in both primary and secondary care, this may be an area that could benefit from further coverage in undergraduate and/or postgraduate medical curricula. Indeed, continued exposure to, and experience in the diagnosis of wound types is essential for the development of practical knowledge related to wound care. Gunay et al., demonstrated that when solely didactic methods are used in undergraduate education, students face significant challenge in the application of theoretical knowledge, which later impacts on individual confidence [12]. Combined didactic and clinical teaching sessions delivered by expert tissue viability nurses may represent a pragmatic approach to improving pre- and post-graduate wound management training.

Our findings are in keeping with studies of other similar interventions. The feasibility and efficacy of mandatory teaching sessions in improving the standard of consent form completion by junior doctors has been demonstrated in a regional orthoplastic hand centre [13].

Postgraduate medical education is limited, and incorporating a new topic, such as wound recognition and dressing selection, will have an opportunity cost, with another topic perhaps being removed. However, identification and examination of surgical wounds and chronic ulcers is pertinent in the management of acutely unwell patients, and one that causes junior doctors a major source of stress in the early stages of their careers [14]. Medical error is a common source of morbidity despite the plethora of safety initiatives, management algorithms and clinical guidance available [15,16]. Limited awareness of local policies, and difficulties in accessing and translating such guidance into clinical practice creates scope for error, reducing patient safety and increasing both direct and indirect healthcare costs. Furthermore, the burden of acute wound assessment and appropriate management falls upon junior doctors, where poor confidence and inappropriate management predispose to wound complications and patient deterioration.

The intervention used here can be deployed quickly and cheaply, without significant expense. Similar initiatives have also been used to increase awareness of evidenced-based guidance and doctors’ confidence in clinical management may help to improve patient safety. Hutton and colleagues demonstrated significant improvement in prescription accuracy, speed and confidence for acute medical conditions using an emergency prescription card containing trust management guidelines for emergency presentations [17].

A limitation of this study is the use of only two formal data collection periods with unpaired data collection pre-and post-intervention due to practical issues. A period of two weeks between intervention and re-audit was chosen to minimise temporary effect and due to the issues of trainees rotating posts. The present body of work was conducted at a single institution with a limited number of study participants which limits the external validity of our findings. The use of a didactic teaching method may not be optimal for the development of pragmatic clinical skill and individual confidence in managing chronic wounds. Further studies should aim to determine whether combined didactic and clinical teaching methods improve knowledge and confidence in the management of chronic wounds.

The repeated use of the same questionnaire may lead to a spurious improvement in performance due to prior exposure to the specific questions at the second administration. However, the FYs were not given the correct answers after the first administration, and we believe that the two-week window between the pre-intervention and post-intervention audits is adequate for washout to have occurred. Despite this, the nature of this study is such that it cannot confirm that true change in clinical practice was achieved, or whether any such change was sustained. Future work involves further liaison with wound care specialists and the tissue viability team to ensure improved access to the Wound Care Formulary and formal inclusion of wound care tutorials into the junior doctor induction programme.

## 5 Conclusion

This quality-improvement study supports a role for a simple and easily reproducible educational intervention to improve wound assessment and dressing selection by FYs. It also increases awareness of a Wound Care Formulary by junior doctors. However, improving FYs confidence in this area may merit further investigation.

### Provenance and peer review

Not commissioned, externally peer reviewed.

## Ethical approval

Ethical approval was not required for this study.

## Sources of funding

No funding was received for the conduct of this study.

## Author contribution

LG, HC & AJG were involved in study design, data collection and analysis. LG, JNR and RZA wrote up the results and concurrent findings.

## Research registration number

researchregistry5314.

https://www.researchregistry.com/browse-the-registry#home/registrationdetails/5e14bc3ff162bf0017a1f463/

## Guarantor

I, Luke Geoghegan, accept full responsibility for the work.

## Declaration of competing interest

The authors declared no potential conflicts of interest with respect to the research, authorship, and/or publication of this article.
